# Surgical Outcome of Ahmed Valve Implantation in Mexican Patients with Neovascular Glaucoma

**DOI:** 10.5005/jp-journals-10008-1168

**Published:** 2015-01-15

**Authors:** Alejandra Hernandez-Oteyza, Gabriel Lazcano-Gomez, Jesus Jimenez-Roman, Carlos Hernandez-Garciadiego

**Affiliations:** Fellow, Department of Glaucoma, Association to Prevent Blindness in Mexico, Mexico; Professor, Department of Glaucoma, Association to Prevent Blindness in Mexico, Mexico; Head, Department of Glaucoma, Association to Prevent Blindness in Mexico, Mexico; Professor, Institute of Mathematics at National Autonomous, University of Mexico, Mexico

**Keywords:** Neovascular glaucoma, Surgical outcome, Ahmed valve implantation, Glaucoma filtration surgery.

## Abstract

**Purpose:** To describe clinical results of Ahmed glaucoma valve implantation in Mexican patients with neovascular glaucoma (NVG).

**Materials and methods:** We reviewed records of 60 eyes of 60 patients with NVG who underwent Ahmed valve implantation, with a follow-up period of 1 year. We identified successful and failed cases and compared baseline and follow-up characteristics to identify possible differences between both groups.

**Results:** We classified 36 eyes (60%) as successful and 24 (40%) as failed cases. We found a significant difference in success rate in patients who had a hypertensive phase at any time during the follow-up period (OR = 5.15, CI = 1.49-20.15, p = 0.004). Patients in the success group showed a statistically significant decrease in the number of glaucoma medications 1 year after surgery (p <0.0001). We found a statistically significant difference in success rate in patients who had preoperative best corrected visual acuity (BCVA) better than logmar 0.70 (odds ratio 4.31, CI = 1.1-19.3, p = 0.03086).

**Conclusion:** A hypertensive postoperative phase and a preoperative BCVA worse or equal to 20/100 seem to be risk factors for Ahmed valve surgical failure in patients with NVG.

**How to cite this article:** Hernandez-Oteyza A, Lazcano-Gomez G, Jimenez-Roman J, Hernandez-Garciadiego C. Surgical Outcome of Ahmed Valve Implantation in Mexican Patients with Neovascular Glaucoma. J Curr Glaucoma Pract 2014;8(3):86-90.

## INTRODUCTION

Glaucoma surgery can be classified as either filtering (increasing outflow) or cyclodestructive (reducing inflow) procedures. Filtration has traditionally been the procedure of first resort because of its efficacy and relative predictability.^1^ Initially, tube shunts were used in eyes with limited visual potential, often in cases where trabeculectomy had already failed, or where trabeculectomy was considered unlikely to succeed. As tube shunts have proven their effectiveness and have shown reduced complication rates compared to traditional filtering surgery, they are being considered more and more as an initial surgical intervention for many types of glaucoma.^2,3^

Neovascular glaucoma (NVG) frequently fails to respond to medical therapy, and trabeculectomy has a high likelihood for failure. Several studies have reported adequate success rates for drainage implants.^4-6^ In a study with the Ahmed glaucoma valve (AGV), the success rate was 68% after an average follow-up of 13 months.^7^ Results of drainage implants in NVG were first reported by Molteno,^8^ but several, mainly retrospective, studies have described the results after Krupin implant, Baerveldt implant and also Ahmed glaucoma valve.^3,4,9,10^ This study aims to evaluate the results of Ahmed glaucoma valve surgery in neovascular glaucoma in a Mexican population.

## MATERIALS AND METHODS

The single-center, retrospective case series was approved by the Institutional Review Board. The consecutive records of patients with NVG due to proliferative diabetic retinopathy who underwent implantation of Ahmed glaucoma valve S-2 (New World Medical Inc., Rancho Cucamonga, California) in 2012 were reviewed. All the surgeries were performed by the same surgeon Gabriel Lazcano-Gomez (GLG). Neovascular glaucoma was defined as neovascularization of the iris and/or anterior chamber angle (NVI/A), with elevated intraocular pressure (IOP)(> 22 mm Hg) that was not responsive to medical therapy with glaucoma medication and previous laser therapy (retina photocoagulation). A minimum follow-up of 1 year after surgery was needed for patients to be included in the study. We excluded patients younger than 18 years old, with previous cyclodestructive procedure or previous glaucoma drainage implant. Patients with no light perception were also excluded.

### Surgical Technique

All patients had subtenon’s anesthesia (2 cc lidocaine 2%) and a fornix-based incision was made through the conjunctiva and Tenon’s capsule with radial relaxing incisions on both sides of the conjunctival flap. Ahmed glaucoma valve implants S-2 were placed into the pocket between the rectus muscles in the superotemporal quadrant. The valves were fixated at 8 to 9 mm posterior to the limbus with a 7 to 0 silk suture on a spatulated needle though the openings on the anterior edge of the plate.

A long needle tract was created with a 23 gauge needle, starting 4 mm behind to the limbus, and viscoelastic was injected into the anterior chamber. The drainage tube was cut bevel up to allow the tube tip to extend approximately 3 mm into the anterior chamber. Conjunctival incisions were closed using the same 7 to 0 silk suture, that were removed 8 to 15 days after the surgery. Topical steroid and antibiotics were started on postoperative day 1 and tapered over the next 8 weeks.

Success was defined as an IOP of > 6 mm Hg and < 21 mm Hg, with or without additional glaucoma medications, without further glaucoma procedures and without loss of light perception. Hypotony was defined as IOP of 5 mm Hg or less on two consecutive visits. A hypertensive phase was defined as IOP > 21 mm Hg during the first 3 months after surgery.

Preoperative baseline information for each patient included: age, sex, best corrected visual acuity (BCVA), IOP, and number of glaucoma medications. After surgery, IOP, number of glaucoma medications, complications and extra surgical procedures on days 1, 7, 30, 60, and months 3, 6 and 12 were registered; BCVA on month 12 was also obtained.

The patients studied were divided into two groups: surgical success group, and surgical failure group, according to the definition previously stated, and both groups were compared to find differences between them.

Data were collected using Microsoft Office Excel 2007 and statistical analysis was done with R (The R Foundation for Statistical Computing, Wirtschaftsuniversitat Wien, Vienna, Austria).^11^

To compare the 2 groups, Mann-Wilcox U test was used for continuous variables and Fisher exact test was used for categorical variables. Shapiro-Wilk, Kolmogorov-Smirnov y QQplot tests were applied to evaluate if IOP values had a normal distribution. Wilcoxon signed rank test was used to compare pre-surgery IOP with IOP values of the postoperative days; p-value less than 0.05 was considered statistically significant.

**Table 1:** **Table 1:** Baseline characteristics

		*Success group* * N = 36 (100%)*		*Failure group* * N = 24 (100%)*		*p*	
Age (y ± SD)		74.42 ± 9.07		75.5 ± 9.47		0.66^Ł^	
Age range		56-88		54-93			
Gender (male : female)		14 (39%) : 22 (61%)		7 (29%) : 17 (71%)		0.44^Ł^	
Eye laterality (right : left)		15 (42%) : 21 (58%)		13 (54%) : 11 (46%)		0.35^Ł^	
Preoperative IPO (mean ± SD) mm Hg		27.06 ± 7.25		29.04 ± 8.76		0.4455*	
Preoperative IPO Range (mm Hg)		17-39		17-46			
Preoperative number of medications (mean ± SD)		3.63 ± 0.54		3.58 ± 0.65		0.73^Ł^	
Preoperative visual acuity BCVA		0.48 ± 0.51		0.85 ± 0.77		0.0068^ŧ^	
Logmar ≤ 1		33 (91%)		19 (79%)			
1 < Logmar ≤ 2		2 (6%)		1 (4%)			
2 < Logmar ≤ 2.3		1 (3%)		4 (15%)			
2.3 < Logmar ≤ 2.7		0		0			
2.7 < Logmar		0		0			

^Ł^t-test; *Mann-Whitney_text; ^ŧ^Mann-Whitney_text (one side)

## RESULTS

A total of 60 patients (60 eyes) were included in the study; 39 were females (65%). Mean age was 74.85 ± 9.09 years (54-93 years). The etiology of the NVG was proliferative diabetic retinopathy in all patients. After 1 year of follow-up, 36 cases (60%) were considered successful and 24 cases (40%) failures. Of the 24 cases considered as failure, 17 eyes (71%) required additional glaucoma surgery and 6 eyes (25%) lost light perception, 7 eyes had hypotension (29%) and 22 eyes had hypertension (91%) in at least one visit. Demographic and baseline characteristics (preoperative data) are shown in Table 1.

Mean preoperative IOP was 27.06 ± 7.25 mm Hg (17-39) for the success group, and 29.04 ± 8.76 mm Hg (17-46) for the failure group showing no significant difference between them (p = 0.4455; by Mann-Whitney test). Mean postoperative IOP was 16.58 ± 2.63 mm Hg (1121) for the success group, and 22.54 ± 6.53 mm Hg (14-37) for the failure group. Both groups had a statistically significant IOP decrease > 15 mm Hg in the first postoperative day (p = 0.0033 and p = 0.0209, respectively; by Wilcoxon signed rank test). All postoperative IOP values were statistically significantly lower when compared to preoperative IOP in both the success group and the failure group (p < 0.0001, p < 0.04, respectively; Wilcoxon signed rank test).

**Table 2:** **Table 2:** Intraocular pressure (12 months follow-up)

		*Success group* *N = 36* *100%*		*Failure group* *N = 24* *100%*		p*	
Preoperative IOP		27.06 ± 7.25		29.04 ± 8.76		0.4455	
IOP (day 1)		7.28 ± 4.1		8.21 ± 4.27		0.2087	
IOP (day 7)		10.94 ± 4.46		10.38 ± 3.75		0.7328	
IOP (month 1)		15.42 ± 5.22		19.71 ± 11.02		0.1476	
IOP (month 2)		18.42 ± 5.54		22.38 ± 8.04		0.0733	
IOP (month 3)		15.22 ± 5.67		20.21 ± 10.01		0.0469	
IOP (month 6)		16.62 ± 3.77		21.58 ± 8.44		0.0396	
IOP (month 12)(final)		16.58 ± 2.63		22.54 ± 6.53		<0.0001	

*p-value using Mann-Whitney text

**Fig. 1:**
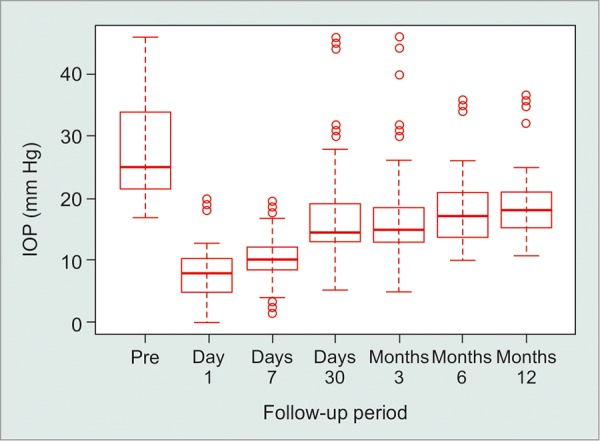
Intraocular pressure (12 months follow-up)

Up to the first 2 months follow-up visits, there was no statistically IOP difference between the success group and the failure group with Mann-Whitney test; however, from the 3rd month until 1st year follow-up visit, there was a statistically significant IOP difference between the two groups (p < 0.05) (Table 2, Fig. 1).

We found no significant difference in success rate between patients who had preoperative IOP > 21 mm Hg and preoperative IOP < 21 mm Hg (odds ratio = 1.45, CI = 0.37-6.34, p = 0.76; by Fisher’s exact test). Nor did we find a significant difference in the success rate in patients who had hypotension on the first day after the surgery (odds ratio = 0.76, CI = 0.19-2.76, p = 0.77; by Fisher’s exact test).

However, we found a significant difference in success rate between patients who had a hypertensive phase at any time during the first year after the surgery *vs* patients with no hypertensive phase (odds ratio = 5.15, CI = 1.49-20.15, p = 0.004; by Fisher’s exact test). All patients who had a hypertensive phase received up to 4 types of topical glaucoma drugs (beta-blockers, alpha-2 agonists, carbonic anhydrase inhibitors and/or prostaglandin analog) as an initial attempt to control the high IOP, and 17 patients in the failure group (71%) required an additional glaucoma surgery (bleb fibrosis removal).

The mean number of preoperative glaucoma medications was 3.64 ± 0.54 in the success group and 3.58 ± 0.65 in the failure group showing no significant difference (p = 0.7321; by t-test). The success group showed a statistically significant decrease in the number of glaucoma medications 12 months after surgery (p < 0.0001; by t-test) but this was not the case in the failure group (p = 0.3724; by t-test). The mean number of medications was statistically significantly lower in the success group compared to the failure group 12 months after surgery (2.02 ± 0.84 *vs* 3.42 ± 2.39, respectively, p = 0.0055; by t-test ‘lower’).

Mean preoperative best corrected visual acuity (BCVA) was 0.48 (logmar) in the success group and 0.85 (logmar) in the failure group; this difference is statistically significant (p = 0.0068; Wilcox signed rank test). At the final visit, BCVA was 0.84 ± 0.66 (logmar) in the success group and 1.51 ± 1.09 (logmar) in the failure group, which represent a 2 and 3-Snellen lines drop, respectively (p = 0.03 *vs* p = 0.017; by t-test). In the success group, 31 patients’ vision (86%) deteriorated, 4 (11°%) patients remained with unchanged vision and 1 (3%) improved it, but less than one Snellen line; in the failure group no patient improved their vision, 4 (17%) maintained it. However, this must be interpreted with caution because 5 patients (13.89%) in the success group and 5 (20.83%) in the failure group required phacoemulsification surgery alone, 6 patients (16.67%) in the success group and 3 (12.50%) in the failure group required phacoemulsification plus vitrectomy and 9 patients (25%) in the success group and 8 (33.33%) in the failure group required vitrectomy alone.

If we consider a BCVA threshold at logmar 0.70 (worse or equal to 20/100) we found a statistically significant difference in success rate between patients who had preoperative BCVA better than 0.70 and those who had worse preoperative BCVA (odds ratio 4.31, CI = 1.1-19.3, p = 0.03086; by Fisher’s exact-test).

**Table 3:** **Table 3:** Complication rate in the failure group

*Complication*		*Eyes*	
		N = 24 (100%)	
Additional glaucoma surgery		17 (71%)	
(bleb fibrosis removal)			
Lost light perception		6 (25%)	
Hypotension		7 (29%)	
Hypertension (at least once during		22 (91%)	
follow-up)			

Postoperative complications included shallow anterior chamber in 8 patients (33.33%) [6 patients (25%) grade 2 and 2 patients (8.33%) grade 3] and hyphema in 4 patients (16.67%). The 2 patients (8.33%) with grade 3 shallow anterior chamber required surgical management of the condition (Table 3).

## DISCUSSION

Neovascular glaucoma is a known risk factor for trabe-culectomy failure, and several studies have shown it to also be a risk factor for glaucoma drainage implant surgery failure.^12^ This study was conducted to evaluate the success rate of Ahmed valve implantation in Mexican patients with NVG.

Although different studies vary in the definition of failure, success rates for Ahmed valve implantation without further interventions after 1 year of follow-up reported in previous studies ranges from 62.5^13^ to 83.8%;^14^ we found a success rate of 60% despite improvement of postoperative IOP. The main cause of surgical failure in other studies has been hypotony,^12^ while we found that 91% of our patients failed due to hypertension and 71% required additional glaucoma surgery.

We found BCVA to drop 2 and 3-Snellen lines in the success and failure groups, respectively, while others have found BCVA to be maintained or improve 1-Snellen line in most patients after treatment.^12,14^ We believe some failure cases can be explained due to the progression of the underlying retinopathy and or the presence of cataract. Ten percent of all our patients (6 of 60) lost light perception, despite initial improvement of IOP; in other studies the proportion of patients with NVG that progressed to no light perception after Ahmed valve implant ranges from 11 to 23.7%.^12,15^

Hyphema has been reported by other authors as the most common complication in up to 35 to 36°%;^16,17^ we found an occurrence of hyphema of 16.67%, but the most common complication found in our patients was shallow chamber, which was observed in 8 patients (33.33%), similar to the findings by Mahdy et al who found an incidence of 30%, while others have not reported this as a predominant complication.^18^

We consider the success rate of glaucoma drainage implant surgery in patients with NVG must be improved by two strategies: first, finding specific risk factors that contribute to failure, and secondly, finding adjunctive treatments that can improve the outcome.

In this study, we found that preoperative IOP and a hypotensive postoperative period do not seem to affect the surgical success rate. However, a hypertensive phase at any time during the first year, does seem to alterthe success rate, so high IOP levels should be treated aggressively.

Our results show that preoperative BCVA also seems to predict a worse outcome with a threshold at logmar 0.70 (worse or equal to 20/100). We believe this is because patients with worse preoperative BCVA have longer time of evolution with NVG when they arrive at the hospital or/and have a worst underlying retinal disease. Thus, NVG must be detected and treated before BCVA decreases more than 20/100 to try and obtain a better result. A limitation to our study is that it did not account for cataracts in the patients.

There have been efforts toward finding a treatment strategy that can improve the visual outcome of patients with NVG. Park et al^14^ compared the success rate and visual preservation of Ahmed valve implantation in patients with and without previous vitrectomy, finding no difference between both groups. Several authors^15,19^ have conducted retrospective studies suggest simultaneous vitrectomy and Ahmed valve implantation via pars plana could be effective in patients with NVG, but further prospective studies are needed to confirm the efficacy of this combined procedure.

Surgical success in cases of NVG treated with intravitreal bevacizumab injection and Ahmed valve implantation have been evaluated by different authors; some have not found benefit in this intervention,^13,17^ while others have.^18,20^ Prospective studies are needed to confirm or rule out such benefit.

Teixeira et al^21^ failed to demonstrate that the intra-vitreal injection of triamcinolone acetonide in patients with NVG affected the success rate of Ahmed valve implantation. Other interventions need to be studied to find a treatment strategy that does improve the success rate in these patients.

Limitations to our study are the relatively short follow-up period and the lack of a comparison group with another indication of Ahmed valve implantation, different to NVG.

Neovascular glaucoma patients require a multi-disciplinary management that includes a retina specialist to control the underling retinopathy and an internal medicine specialist to control the underling systemic cause (that is diabetes mellitus).

## CONCLUSION

In Mexican patients with NVG treated with Ahmed valve, a postoperative hypertensive phase and a preoperative BCVA worse or equal to 20/100 seem to be risk factors for surgical failure. Further studies are needed to find other risk factors associated with failure and other therapeutic strategies to improve success.
